# A manifold neural population code for space in hippocampal coactivity dynamics independent of place fields

**DOI:** 10.1016/j.celrep.2023.113142

**Published:** 2023-09-25

**Authors:** Eliott Robert Joseph Levy, Simón Carrillo-Segura, Eun Hye Park, William Thomas Redman, José Rafael Hurtado, SueYeon Chung, André Antonio Fenton

**Affiliations:** 1 Center for Neural Science, New York University, New York, NY 10003, USA; 2 Graduate Program in Mechanical and Aerospace Engineering, Tandon School of Engineering, New York University, Brooklyn, NY 11201, USA; 3 Interdepartmental Graduate Program in Dynamical Neuroscience, University of California, Santa Barbara, Santa Barbara, CA 93106, USA; 4 Flatiron Institute Center for Computational Neuroscience, New York, NY 10010, USA; 5 Neuroscience Institute at the NYU Langone Medical Center, New York, NY 10016, USA; 6 These authors contributed equally; 7 Lead contact

## Abstract

Hippocampus place cell discharge is temporally unreliable across seconds and days, and place fields are multimodal, suggesting an “ensemble cofiring” spatial coding hypothesis with manifold dynamics that does not require reliable spatial tuning, in contrast to hypotheses based on place field (spatial tuning) stability. We imaged mouse CA1 (cornu ammonis 1) ensembles in two environments across three weeks to evaluate these coding hypotheses. While place fields “remap,” being more distinct between than within environments, coactivity relationships generally change less. Decoding location and environment from 1-s ensemble location-specific activity is effective and improves with experience. Decoding environment from cell-pair coactivity relationships is also effective and improves with experience, even after removing place tuning. Discriminating environments from 1-s ensemble coactivity relies crucially on the cells with the most anti-coactive cell-pair relationships because activity is internally organized on a low-dimensional manifold of non-linear coactivity relationships that intermittently reregisters to environments according to the anti-cofiring subpopulation activity.

## In brief

Levy et al. find seconds-timescale hippocampus population discharge is relatively unchanged across environments despite place fields remapping to random locations. This population cofiring defines a CA1 manifold that distinctly registers to each environment by an environment-specific anti-cofiring subset of cells that mainly discharge when most other cells do not.

## Graphical abstract

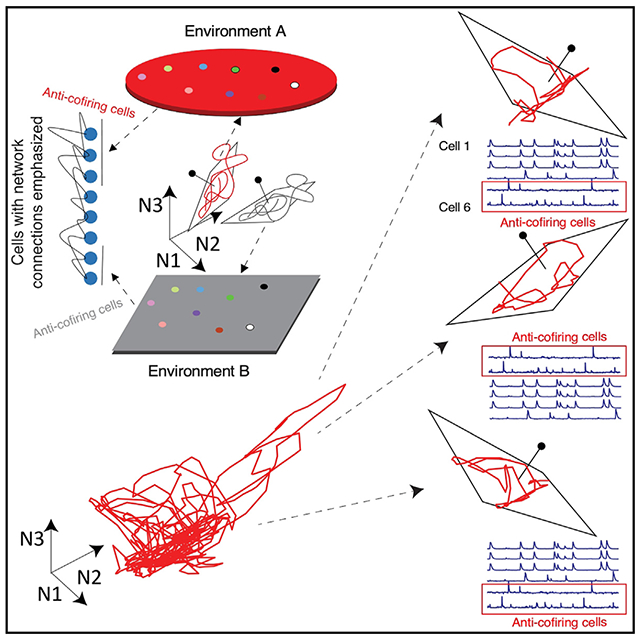

## INTRODUCTION

**Figure 1.**
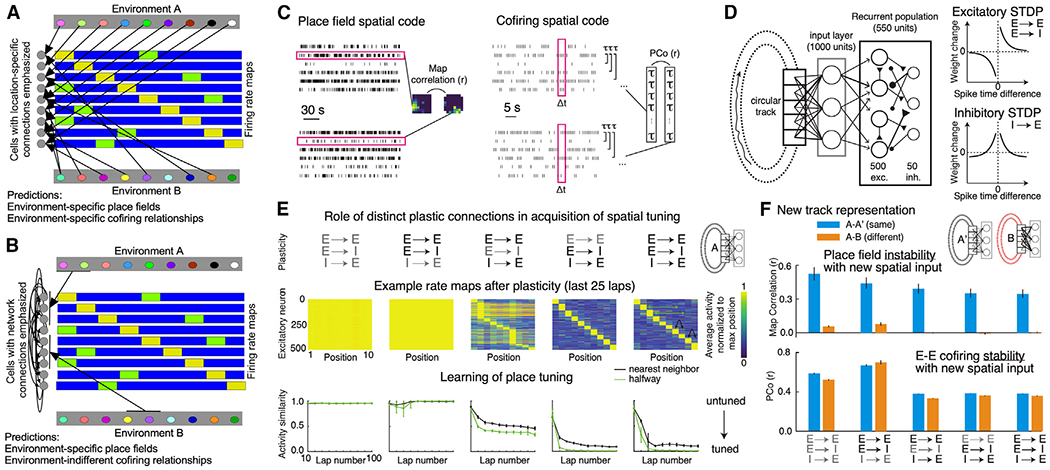
Neural coding hypotheses for representing environments (A and B) Illustration of (A) the concept of remapping, emphasizing that cell-specific position inputs change place field locations and ensemble cofiring relationships across environments, and (B) the concept of reregistration, emphasizing intrinsic hippocampal inputs that do not change between environments despite changing network registration to environmental cues. Linearized firing-rate maps depict environment-specific multipeaked place fields (yellow and green). (C) Dedicated place field (left) and ensemble cofiring (right) coding hypotheses and their analyses by single-cell spatial firing stabilities or by the stability (PCo^10^) of the n(n-1)/2 Kendall correlations (τ), respectively. (D) Schematic neural network encoding a ring environment with STDP learning rules. (E) Learned ring representation with/without (black/gray) STDP learning at cell-type-specific connections (top), quantified by rate maps (center) and spatial activity similarity (bottom). The nearest-neighbor metric measures the ratio of activity at the peak and neighboring locations; the halfway metric measures the ratio of activity at the peak and activity at the position halfway across the track. (F) Remapping the external inputs to the network (track B) compared with an unchanged mapping (null remapping, track A′) and evaluation of (top) dedicated place field and (bottom) ensemble cofiring codes in track A and B environments with/without (black/gray) STDP learning at cell-type specific connections after changing the inputs. Errror bars: SEM.

The place fields of each hippocampus place cell change uniquely between environments, a phenomenon called “remapping.” The original concept emphasized a rearrangement of temporal cofiring relationships relating neural activity to the environment,^1–6^ but most studies infer a temporal rearrangement from changed time-normalized place fields of individual cells. However, cofiring can change without changing place fields,^7^ and place fields can change without changing short-timescale cofiring relationships with other cells, especially when place cells have multiple non-periodic place fields.^8,9^ Furthermore, remapping implies that place fields are determined by environmental features (Figure 1A),^5,11–15^ but place fields would also rearrange when only the registration changes between the environment and a subpopulation of invariant cofiring relationships that are internally organized (Figure 1B).

Because memory and context representations rely on the hippocampus, the dominant hypothesis asserts that remapping and memory are intimately linked; changes in place tuning are assumed to correspond to changes in memory, especially episodic representations.^1,16–21^ Studies designed to relate remapping to memory have been remarkably unsupportive,^22–26^ nor is it straightforward how the arrangement of place fields can represent a particular environment at the millisecond-to-second timescale of neural computations without initial, extensive spatial exploration,^27^ and for additional fundamental reasons: (1) multimodality, multiple place fields in environments bigger than ~1 m^2^;^8,9^ (2) overdispersion, discharge is extremely variable during the 1–5 s of crossing a place field;^28–31^ (3) rate remapping, place field firing rates vary across behavioral episodes;^19,32^ (4) mixed selectivity, discharge encodes multiple variables;^33,34^ and (5) instability, a minority of place fields are stable across days in familiar environments.^35,36^ Multimodality alone degrades decoding environments from ensemble discharge (Figure S1).

Might standard assumptions about how neural information is encoded be incorrect? We standardly average the activity of individual cells over minutes, discarding temporal discharge fluctuations and cofiring relationships with other cells to extract the cell’s discharge relationship to any variable. Such place cell analyses incorrectly assume steady-state place-discharge relationships.^28–30,37,38^ This standard dedicated-rate place field hypothesis assumes that the cell’s momentary firing rate independently carries information that can be adequately extracted by analysis of each cell’s place tuning. The firing relations of each cell to other neurons are assumed to be uninformative, or at least secondary, and are ignored by data representations like firing rate maps.

While it is intuitive to imagine that cells with overlapping place fields also cofire because time and space are confounded, some place cell pairs with overlapping place fields reliably cofire, while other pairs do not, and other pairs discharge independently on the milliseconds-to-seconds timescales of crossing firing fields and neural computation (Figure S3).^39–41^ This motivates an alternative cofiring coding hypothesis that asserts that information is encoded in the momentary cofiring patterns among large groups of neurons—each cofiring pattern may involve cells with no place field or mixed selectivity.^33,41,42^ Such cofiring neural codes explicitly recognize that high-dimensional neuronal population activity can be constrained to manifolds, low-dimensional subspaces defined by recurring ensemble patterns of coactivity.^43–54^ These cofiring patterns are internally organized; they can be established early in experience and expressed independent of particular environmental stimuli.^55–59^ Higher-order firing patterns constrained on a manifold of particular topology and geometry can, without changing shape, describe infinitely many individual cell discharge patterns and even an infinity of lower-order cofiring patterns,^46,53,60^ as demonstrated by medial entorhinal cortex (MEC) grid cells.^45,54^ Such discharge patterns are constrained by “on-manifold” cofiring relationships, and because they need not ever repeat, they need not depend on individual tuning to external variables like locations, which contrasts with the standard view that the variability in neuronal activity comes from variability in behavior, as demonstrated in flying bats,^61^ despite the firing rate overdispersion that is observed in rodents, even during taxing memory tasks.^23,28–31,62,63^ We repeated a standard place cell remapping procedure to evaluate the dedicated place field and ensemble cofiring hypotheses for representing distinct environments in the hippocampus.

## RESULTS

### Distinguishing place tuning and cofiring contributions to representing environments: A neural network model

We examined a heuristic neural network model to evaluate contributions of place tuning (Figure 1C, left) and cofiring (Figure 1C, right) to representing environments. The recurrently connected excitatory (E)-inhibitory (I) network receives E input from units tuned to random positions of a ring track (Figure 1D, left). The recurrent weights change according to standard spike-timing-dependent plasticity (STDP) rules (Figure 1D, right) that are sufficient to generate place fields (Figure 1E, center) after ~30 laps (Figure 1E, bottom). E→I and I→E but not E→E plasticity is necessary for the place tuning (Figure 1E), reproducing observations after blocking NMDA (N-methyl-D-aspartate) receptor-dependent long-term potentiation(LTP),^7,64,65^ which dominates at E→E but not at E→I or I→E hippocampal connections.^65^ This illustrates that tunable excitation-inhibition coordination can be important for network and memory function, as observed previously.^23,66–72^ To simulate remapping, we randomized the position-to-input relationships and allowed STDP changes of the recurrent weights for 30 laps. The place fields reorganized and were unrelated across the two tracks (Figure 1F, top; Figure S2A). E-E cofiring was relatively unchanged Figure S2 (Figure 1F, bottom) but marginally more stable when spatial inputs were unchanged compared with changed. Cofiring changes with experience, regardless of whether the environmental inputs change, mostly because of I→E plasticity. This heuristic demonstrates that several ideas are feasible: (1) changing place fields between environments may occur without changing most E-E cofiring relationships; (2) place field locations depend on the environment-specific anti-cofiring E-E relationships, a consequence of necessarily altered I→E (and E→I) functional connections, which have been observed as experience-dependent^68^ and learning-dependent changes in network inhibition;^71^ and (3) remapping may be a misnomer for what is a reregistration between a neurocentric, internally rigid neural activity representation and external features.

### Calcium imaging of CA1 activity

**Figure 2.**
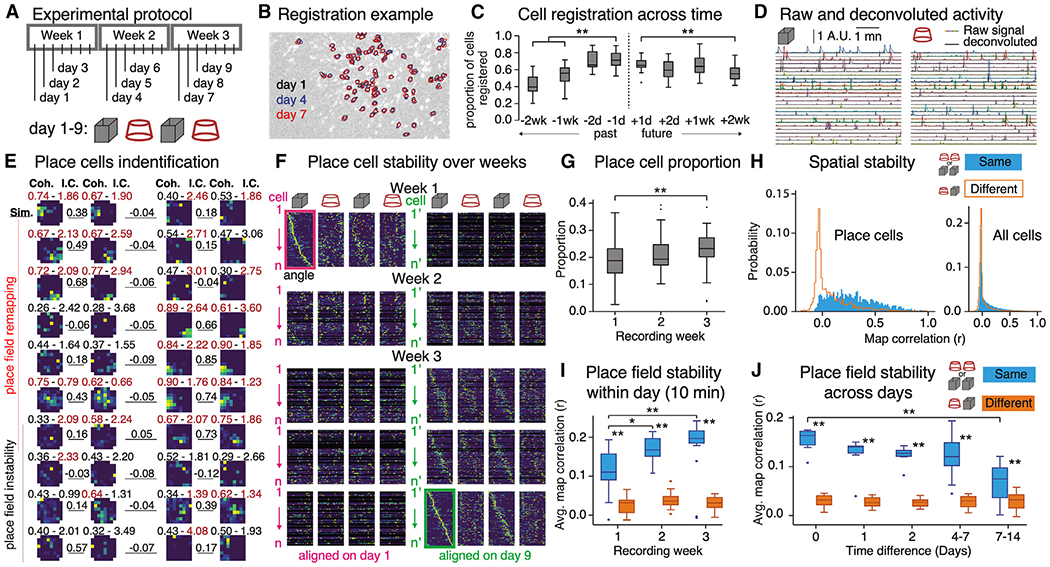
Location-specific CA1 activity imaged across weeks is transient and describes a minority of the CA1 population, and firing fields remap between environments (A) Experimental protocol. (B and C) Example (B) and quantification (C) of cell registration across weeks (past: time F_3,187_ = 56.89, p = 10^−26^, $\eta^{2}=0.48$; Dunnett’s tests: −1 day > −2, −1 week(s); future: time F_3,187_ = 7.31, p = 10^−4^, $\eta^{2}=0.12$; Dunnett’s tests: 1 day >2 weeks). (D) Activity time series in two environments of a 25-cell subsample from a 546-cell ensemble. (E) Example rate maps from a 588-cell ensemble across two environments on recording day 7. Place field quality metrics, spatial coherence (Coh.; left), and information content (I.C.; right) are indicated above each map (red significant compared with shuffled); average rate map similarity (Sim.; r) is indicated between maps. (F) Ensemble of linearized rate maps across weeks (week 1: day 1; week 2: day 4; week 3: days 7, 8, 9), matched and aligned to activity on recording day 1 (left, 312 cells) and recording day 9 (right, 460 cells). (G) Place cell prevalence (week: F_2,64_ = 43.70, p = 10^−12^, $\eta^{2}=0.085$; Dunnett’s tests: week 1 < week 3). (H) Place map similarity across environments on weeks 2 and 3 illustrates remapping of place fields (left) but not the CA1 population (right). (I) Within-day place map similarity improves across weeks (week: F_2,28_ = 20.02, p = 10^−6^, $\eta^{2}=0.051$; env. change: F_2,29_ = 80.55, p = 10^−10^, $\eta^{2}=0.65$; interaction: F_2,28_ = 4.83, p = 0.016, $\eta^{2}=0.026$; Dunnett’s tests: week 1 < weeks 2, 3 for similar environments; post hoc: same vs. different is significant each week; **p ≤ 10^−5^). (J) Place map similarity degrades across days (time difference: F_4,132_ = 12.68, p = 10^−9^, $\eta^{2}=0.11$; env. change: F_1,132_ = 228.37, p = 10^−30^, $\eta^{2}=0.53$; interaction: F_4,132_ = 9.32, p = 10^−6^, $\eta^{2}=0.12$; Dunnett’s tests: day 0 > 7–14 days for similar environments; post hoc: same vs. different is significant at each interval, **p ≤ 10^−3^). For all boxplots, the height is determined by the interquartile range, and the median is indicated with a line. The whiskers extend to the furthest data point up to 1.5 times the interquartile range past the boxplot limit. Data points past the whisker limits are either plotted individually or omitted when too large for better visual representation. Statistically significant differences are indicated on the plots. A statistically significant effect of the factor week is indicated as a line above the graph with asterisk(s). One asterisk indicates p < 0.05 (or the corresponding corrected value) and two asterisks indicate p < 0.01 (or the corresponding corrected value).

CA1 was infected with AAV-GCaMP6f,^73,74^ and CA1 ensemble activity was recorded with a miniature microscope (http://miniscope.org)^74^ placed over a chronically implanted gradient index (GRIN) lens.^35^ The mice (n = 6) were habituated to the miniscope in their home cages, and during the recordings, they explored a cylinder and box (Figure 2A). Raw calcium traces were extracted and segmented from videos using the constrained nonnegative matrix factorization for microendoscopic data (CNMF-E) algorithm^75,76^ and registered to individual cells across the 3-week protocol (Figure 2B). Over 50% of individual cells could be identified 2 weeks apart, though the registration was greater across shorter intervals (Figure 2C). Raw calcium traces were converted with the CNMF-E algorithm to spiking activity for analysis (Figure 2D).

### Place field measurements of remapping

Place fields were stable within a day and unstable across days, as reported previously (Figure 2E).^35,36^ Each environment was linearized and individual activity rates plotted as a function of angle (Figure 2F). The location-specific pattern expressed in week 3 (aligned on recording day 9) was observed during week 2, but not the pattern from week 1 (aligned on recording day 1), suggesting that place tuning improved during the first week. Place cell prevalence increased with experience from 20% to 25% (Figure 2G).^33,77^ Each day, place cells expressed stable firing fields in the same environment, but location specificity changed between different environments (Figure 2H, left). Such remapping is not population wide because the measurable difference vanishes when all recorded cells are considered. This is largely because the place tuning of non-place cells is unstable between any pair of trials (Figure 2H, right). The average within-day place field stability of place cells grows with experience from 0.1 during week 1 to 0.2 during week 3 (Figure 2I), about 6 times larger than for distinct environments on week 3. This place field stability degrades across days (Figure 2J), as described previously.^35,36^ These measurements demonstrate standard place cell phenomena.

### Ensemble cofiring measurements between environments

**Figure 3.**
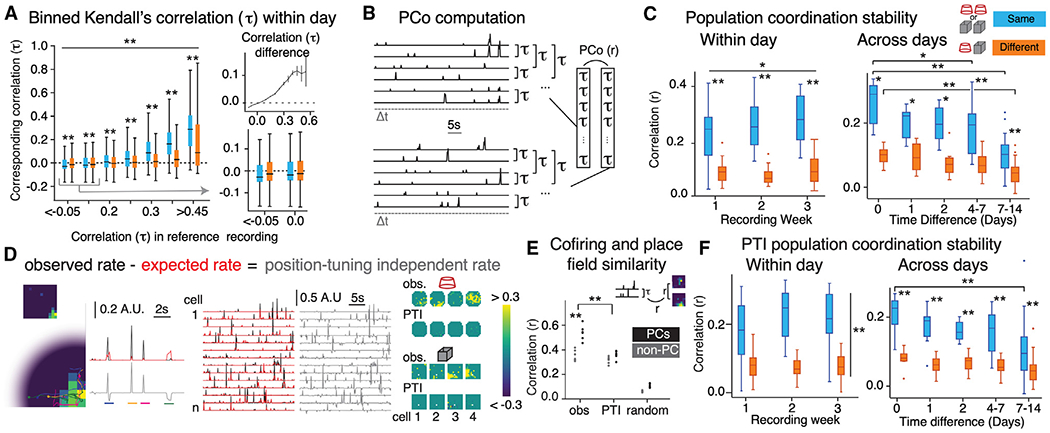
Ensemble cofiring distinguishes two environments independent of place tuning (A) Comparison of coactivity (τ) stability of all cell pairs, from weeks 2 and 3, across same-day recordings, for different initial coactivity values. Coactive cell pairs tend to remain coactive across the same but not different environments (coactivity level: F_6,3285501_ = 9,825.46, p = 0.00, $\eta^{2}=0.018$; env. change: F_1,3285501_ = 9.82, p = 0.0017, $\eta^{2}=10^{-6}$; interaction: F_6,3285501_ = 3,395.58, p = 0.00, $\eta^{2}=0.0061$; post hoc: same vs. different is significant at each coactivity level, **p < 10^−15^) and (bottom inset) anti-coactive cell pairs remain anti-coactive. Top inset: average signed differences confirm the effect of environment change. (B) Illustration of population coordination (PCo) calculation. One-second time intervals (Δt=1s) used to discretize activity are illustrated as gray dashes, to scale. (C) Left: within-day coactivity stability improves across weeks (week: F_2,27_ = 4.23, p = 0.025, $\eta^{2}=0.015$; env. change: F_1,28_ = 92.32, p = 10^−10^, $\eta^{2}=0.56$; interaction: F_2,27_ = 0.99, p = 0.38, $\eta^{2}=0.022$; post hoc: same vs. different is significant each week, **p < 10^−4^). Right: across-day cofiring stability degrades over time (time difference: F_4,132_ = 16.79, p = 10^−11^, $\eta^{2}=0.21$; env. change: F_1,132_ = 94.66, p = 10^−17^, $\eta^{2}=0.32$; interaction: F_4,132_ = 4.04, p = 0.004, $\eta^{2}=0.072$; Dunnett’s tests vs. day 0, *p < 0.0058, **p ≤ 10^−5^; post hoc: same vs. different is significant at each time interval, **p ≤ 10^−3^). (D) PTI (left to right) example activity map and magnified version with four passes across the place field (star = path start). Shown are observed, expected, and PTI activity time series with the color-coded passes marked and additional examples and observed and PTI activity maps from four example cells. (E) Place field similarity and coactivity correlations, computed from observed, PTI, and randomized PTI activities, for place cells and non-place cells (time series type: F_2,30_ = 315.13, p = 10^−21^, $\eta^{2}=0.84$; functional cell class: F_1,30_ = 56.74, p = 10^−8^, $\eta^{2}=0.076$; interaction: F_2,30_ = 14.72, p = 10^−5^, $\eta^{2}=0.039$; post-hoc: PC vs. non-PC for observation (obs) and obs vs. PTI for place cells significant, **p ≤ 10^−4^). (F) Left: within-day PTI coactivity stability across weeks (week: F_2,27_ = 2.28, p = 0.12, $\eta^{2}=0.009$; env. change: F_1,28_ = 46.67, p = 10^−7^, $\eta^{2}=0.52$; interaction: F_2,27_ = 2.38, p = 0.11, $\eta^{2}=0.028$; post hoc: same vs. different: t_67.44_ = 10.14, p = 10^−15^). Right: across-day PTI coactivity stability also degrades over time (time difference: F_4,132_ = 5.23, p = 10^−4^, $\eta^{2}=0.088$; env. change F_1,132_ = 66.57, p = 10^−13^, $\eta^{2}=0.33$; interaction: F_4,132_ = 1.69, p = 0.16, $\eta^{2}=0.079$; Dunnett’s tests: day 0 > days 7–14 for similar environments, **p = 0.0050; post hoc same vs. different significant at each time interval, **p < 0.008). For all boxplots, the height is determined by the interquartile range and the median is indicated with a line. The whiskers extend to the furthest data point up to 1.5 times the interquartile range past the boxplot limit. Data points past the whisker limits are either plotted individually or omitted when too large for better visual representation. Statistically significant differences are indicated on the plots. A statistically significant effect of the factor on the x-axis is indicated as a line above the graph with asterisk(s). One asterisk indicates p < 0.05 (or the corresponding corrected value) and two asterisks indicate p < 0.01 (or the corresponding corrected value).

Next, we examined the change in CA1 activity across the two environments, ignoring positional information. In each trial, for each cell pair, we measured the coactivity of their 1-s activity time series by computing Kendall’s correlation (Figure 3A), which estimates the recurrence of higher-order correlations, responsible for the ensemble’s characteristic population geometry (Figure S3).^78^ To evaluate the cofiring stability across environments, we measured the population coordination (PCo) as the Pearson correlation between two vectors of paired τ values Figure 3B.^10,79^ PCo between trials of the same environments is three times higher than between distinct environments (Figure 3C), indicating that ensemble coactivity discriminates environments. The cell pairs that differ the most between environments are the positively (τ>0.3) or negatively (τ<−0.05) coactive cell pairs (Figure 3A). PCo increases with experience (Figure 3C, left) and decreases with greater time between recordings (Figure 3C, right), like place field measurements (Figures 2I and 2J). These findings indicate that environments are discriminatively encoded in the 1-s coactivity relationships of CA1 cells.

### Ensemble cofiring measurements of remapping between environments independent of position tuning

We evaluated the extent to which position tuning explains the 1-s coactivity relationships by calculating a position-tuning independent (PTI) rate. We subtracted the expected rate from the observed rate (Figure 3D), the expected rate at the current position corresponding to the rate in the session-averaged firing rate map.^28,29^ Place fields computed from the PTI rate disappear (Figure 3D, right). Nonetheless, correspondence is high (0.4 < r < 0.6) between cell-pair correlations computed from the observed rates of the cell pairs and those computed from the PTI rates, indicating activity correlations in cofiring beyond position tuning (Figure S4B), as also observed ubiquitously in the neocortex with cells tuned to different variables.^80–83^

Cofiring computed from either observed or PTI rates covaries with spatial firing similarity for place cells and non-place cells (Figure 3E). Taken together, this indicates that place fields are responsible for a small fraction of the relationship between cell-pair correlation and spatial firing similarity (Figure S4). PCo computed from PTI (PTI-PCo) decreases by ~20%, remains higher for comparisons between the same environment compared with between different environments, but does not change from week 1 to week 3 (Figure 3F, left). Like PCo computed from observed rates (Figure 3C, right), PTI-PCo also degrades over time (Figure 3F, right).

### Decoding environments from PTI ensemble coactivity

**Figure 4.**
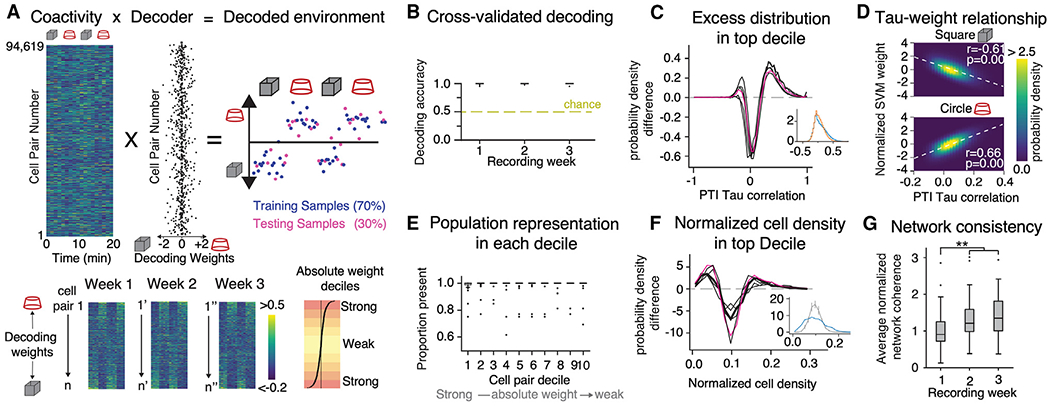
Decoding environments from PTI coactivity (A) SVM decoding from PTI coactivity. Top: PTI coactivity, computed every 60 s, is projected with SVM weights onto a single classification dimension. Bottom: cell-pair coactivity from an example ensemble ordered by SVM weight each week; weights’ absolute values are classified by decile. (B) Decoding accuracy for all ensembles. (C and D) Difference between overall and first decile distribution of cell-pair correlations (C, inset: purple’s component distributions) indicates the importance of coactive and anti-coactive cell pairs, confirmed by (D) 2D distribution of SVM weights against PTI coactivity. (E) Proportion of individual cells present in at least one cell pair from each SVM-ordered decile. (F) Difference between random and first decile, normalized distributions of individual cell participation (inset: purple’s component distributions). (G) PTI network consistency normalized by the standard deviation of the cell-pair randomized distribution (week: F_2,60_ = 15.76, p = 10^−6^, $\eta^{2}=0.094$; Dunnett’s tests: week 0 < weeks 2, 3). For all boxplots, the height is determined by the interquartile range and the median is indicated with a line. The whiskers extend to the furthest data point up to 1.5 times the interquartile range past the boxplot limit. Data points past the whisker limits are either plotted individually or omitted when too large for better visual representation. Statistically significant differences are indicated on the plots. A statistically significant effect of the factor on the x-axis is indicated as a line above the graph with two asterisks indicating p < 0.01 (or the corresponding corrected value).

We then investigated whether the current environment could be reliably decoded from the PTI ensemble coactivity. Coactivity was computed at 1-s resolution during 1 min. A support vector machine (SVM) decoder (Figure 4A) almost always correctly identifies the current environment (Figure 4B), demonstrating that coactivity itself carries discriminative information. The most discriminative cell pairs are either strongly coactive or strongly anti-coactive (Figures 4C and 4D). Most individual cells contributed to cell pairs in each discriminative decile (Figure 4E), but some cells were overrepresented in the subset of discriminative cell pairs that constituted the first decile (Figure 4F). Activity correlations are scale-free because power laws fit their distribution as a function of distance between the somata; best-fit exponents are negative and slightly smaller than 1 (−0.90 ± 0.05; Figure S4D). Accordingly, correlation length (the distance at which average correlation = 0) is large; on average, the activity of each cell non-causally influences the activity of every other cell. The environment-discriminating activity of the neural population is driven by a subset of the cells but impacts the whole population, reminiscent of flocking and schooling of birds and fish.^84–86^ Network consistency,^23^ which estimates the overall correspondence between cell pair (*i* and *j*) PTI coactivity ($\tau_{i,j}$) and the pair’s instantaneous PTI rate (*r_i_* and *r_j_*), increases with experience (Figure 4G), indicating an increased alignment of the 1-s short and 300-s long timescales of cofiring in the network. Repeating the set of coactivity calculations without first eliminating the place fields yielded similar but less discriminative results (Figure S4; week: F_2,19_ = 34.96, p = 10^−7^, $\eta^{2}=0.023$; time series type: F_1,20_ = 14.66, p = 0.001, $\eta^{2}=0.35$; interaction: F_2,19_ = 26.61, p = 10^−6^, $\eta^{2}=0.011$; post hoc: PTI > observed on weeks 1 and 3, p ≤ 0.004; p = 0.027 on week 2). Parenthetically, decoding current location from 1-s place cell activity vectors is somewhat better than decoding from 1-s cofiring assessed by coincidence (Figure S6).

### The environment-discriminating subset of CA1 cells

**Figure 5.**
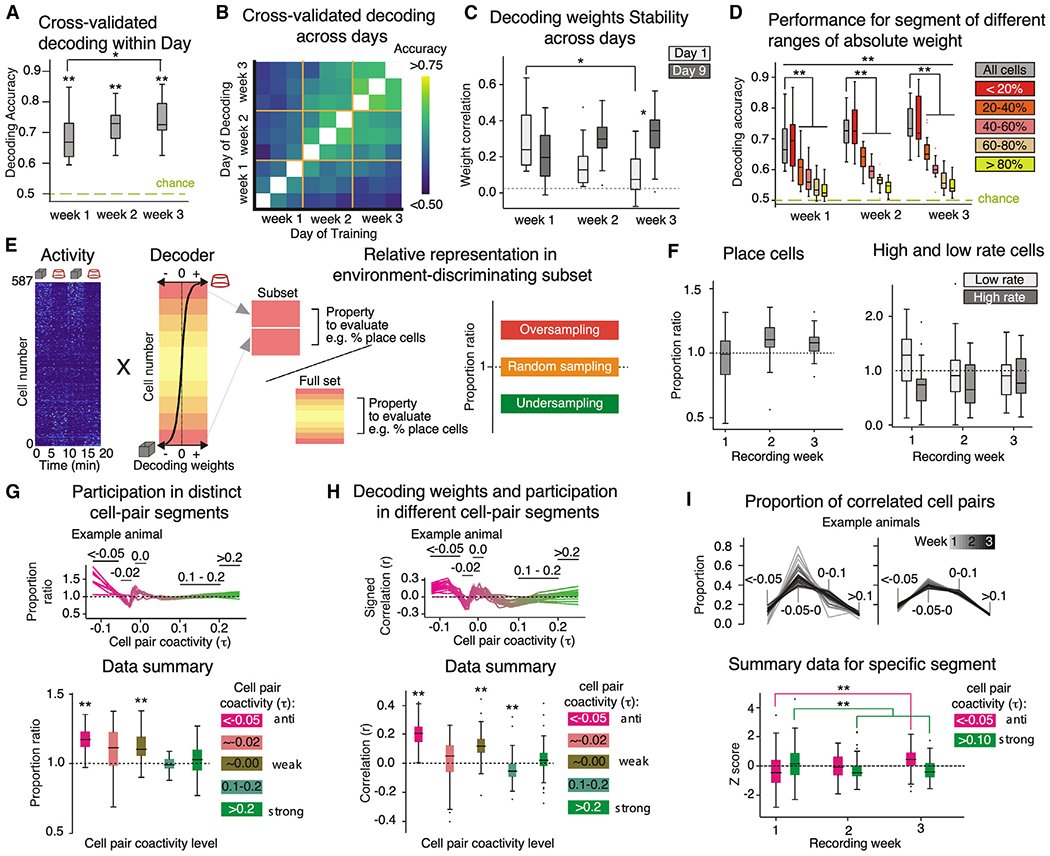
Decoding 1-s activity vectors relies on anti-coactive cell pairs (A) Decoding each week (week: F_2,12_ = 12.63, p = 0.0011, $\eta^{2}=0.11$; Dunnett’s tests: week 1 < week 3; post hoc t tests vs. 0, **p < 10^−7^). (B) Heatmap of decoding performance across days. (C) Correlation of weights trained on days 1 and 9 with weights trained on other days, with weights from the same day excluded (no effect of week: F_2,9_ = 2.11, p = 0.18, $\eta^{2}=0.011$; no effect of day: F_1,10_ = 1.71, p = 0.22, $\eta^{2}=0.08$; interaction: F_2,9_ = 8.03, p = 0.01, $\eta^{2}=0.14$; Dunnett’s tests: week 0 < week 2 on day 1; post hoc t tests: day 1 vs. day 9, *p < 0.017). (D) Cells are ordered by SVM absolute weights and separated into quintiles (20%) whose cross-validated decoding is measured independently (quintile: F_4,65_ = 38.58, p = 10^−16^, $\eta^{2}=0.65$; week: F_2,64_ = 29.86, p = 10^−10^, $\eta^{2}=0.022$; interaction: F_8,89.135_ = 3.69, p = 10^−4^, $\eta^{2}=0.026$; post hoc t tests: all cells vs. quintiles, **p ≤ 0.001; Dunnett’s tests: week 0 < week 3 for quintiles 1 and 2). (E) Left to right: activity vectors with cells ordered by SVM weights. Proportion ratio: (i) weights’ absolute value classified by quintiles. (ii) compute ratio of the environment-discriminating subset’s decoding (top quintile) relative to the population’s. (F) Proportion ratio of cells classified as (left) a place cell in either environment (no effect of week: F_2,12_ = 3.58, p = 0.061, $\eta^{2}=0.13$; t test vs. unity [1]: t_48_ = 1.38, p = 0.18) and (right) high or low rate (high rate: no effect of week: F_2,12_ = 0.65, p = 0.54, $\eta^{2}=0.015$;t test vs. unity [1]: t_48_ = 3.19, p = 0.0025; low rate: no effect of week: F_2,12_ = 3.47, p = 0.065, $\eta^{2}=0.064$; t test vs. 1:t_48_ = −0.07, p = 0.95). (G) Proportion ratio for different coactivity levels (top) example, (bottom) dataset (coactivity level: F_4,65_ = 5.71, p = 10^−4^, $\eta^{2}=0.094$; no effect of week: F_2,65_ = 0.19, p = 0.83, $\eta^{2}<10^{-3}$; no interaction: F_8,128_ = 1.14, p = 0.34, $\eta^{2}=0.094$; post hoc t tests vs. 1, **p ≤ 10^−6^). (H) Correlations between SVM weight and participation in cell pairs of different coactivity levels (top) example, (bottom) dataset (coactivity level: F_4,135_ = 55.53, p = 10^−27^, $\eta^{2}=0.43$; no effect of week: F_2,134_ = 1.57, p = 0.21, $\eta^{2}=0.0012$; no interaction: F_8,189.12_ = 1.82, p = 0.076, $\eta^{2}=0.018$; post hoc t tests vs. 0, **p ≤ 10^−7^). (I) Proportion of cell pairs with different coactivity levels across days (top) two examples, (bottom) cofiring and anti-cofiring cell pairs (no effect of coactivity level: F_1,122_ = 0.74, p = 0.39, $\eta^{2}=0.0043$; week: F_2,121_ = 3.91, p = 0.023, $\eta^{2}=0.0075$; interaction: F_2,121_ = 10.12, p = 10^−5^, $\eta^{2}=0.07$; Dunnett’s tests vs. week 1, **p ≤ 0.009). For all boxplots the height is determined by the interquartile range and the median is indicated with a line. The whiskers extend to the furthest data point up to 1.5 times the interquartile range past the boxplot limit. Data points past the whisker limits are either plotted individually or omitted when too large for better visual representation. Statistically significant differences are indicated on the plots. A statistically significant effect of the factor on the x-axis is indicated as a line above the graph. with asterisk(s). One asterisk indicates p < 0.05 (or the corresponding corrected value) and two asterisks indicate p < 0.01 (or the corresponding corrected value).

CA1 population activity vectors are distinct between the two environments, but this difference is remarkably small (Figure S7), consistent with the notion that the activity of only a minority of cells contributes to discriminating the environments. Using 1-s activity vectors recorded on the same day, the SVM decoder correctly identifies the environment well above chance; performance increases across weeks (Figure 5A). After 1 week, decoding across days also performs above chance (Figure 5B). Indeed, after 1 week, the SVM weights are stable across weeks; the weights obtained from the last day of the third week are strongly correlated with weights up to 9 days earlier, suggesting that SVM weight stability reflects learning (Figure 5C). To evaluate which subset of cells contributes to discriminating the environments, we trained a decoder using select portions of the population to which the initial SVM decoder gave different weights. Decoders using the 20% of cells with the largest weights perform indistinguishably from decoding with the entire population. On the other hand, decoders using 40% of cells with the smallest SVM weights perform close to chance (Figure 5D). Decoders using cells with the 20% highest weights also display the largest increase in performance with experience, while for the bottom 60%, performance does not increase, consistent with the expectation that the largest SVM weights estimate the strength of spatial coding and learning, much like place field quality has been interpreted.

We call this 20% of cells the “environment-discriminating subset” and evaluated their properties by computing whether the properties of cells in the subset are more or less prevalent than in the entire population (Figure 5E). The place cells, strongly active cells, and weakly active cells are not more likely than chance to be in the environment-discriminating subset (Figure 5F). Only the anti-coactive cells are more likely than chance to be part of the environment-discriminating subset (Figure 5G). Furthermore, the SVM decoding weights are predicted by the number of negatively correlated cell pairs to which a cell belongs. By contrast, participation in positively correlated pairs is not related to either SVM weights or being in the environment-discriminating subset (Figure 5H). The number of negatively correlated cell pairs increased, and the number of positively correlated pairs decreased, with discriminating the two environments (Figure 5I). We visualized this by computing each cell’s anti-cofiring power (proportion of significantly negative correlations τ≤−0.05). Figure S8 shows ensembles recorded in weeks 1 and 3 as matrices of the cell-pair correlations. The row 2 and 3 matrices are organized according to increasing anti-cofiring power determined in one environment. The anti-cofiring subset appears to be more stable after experience, consistent with learning (Figure 5I). The most anti-cofiring cells can even be anti-correlated with each other, and the anti-cofiring subset is environment specific, with up to 40% overlap between the anti-cofiring subset of cells in the same environment on day 9 (Figure S8C).

### Planar manifold topology of ensemble firing distinguishes environments

**Figure 6.**
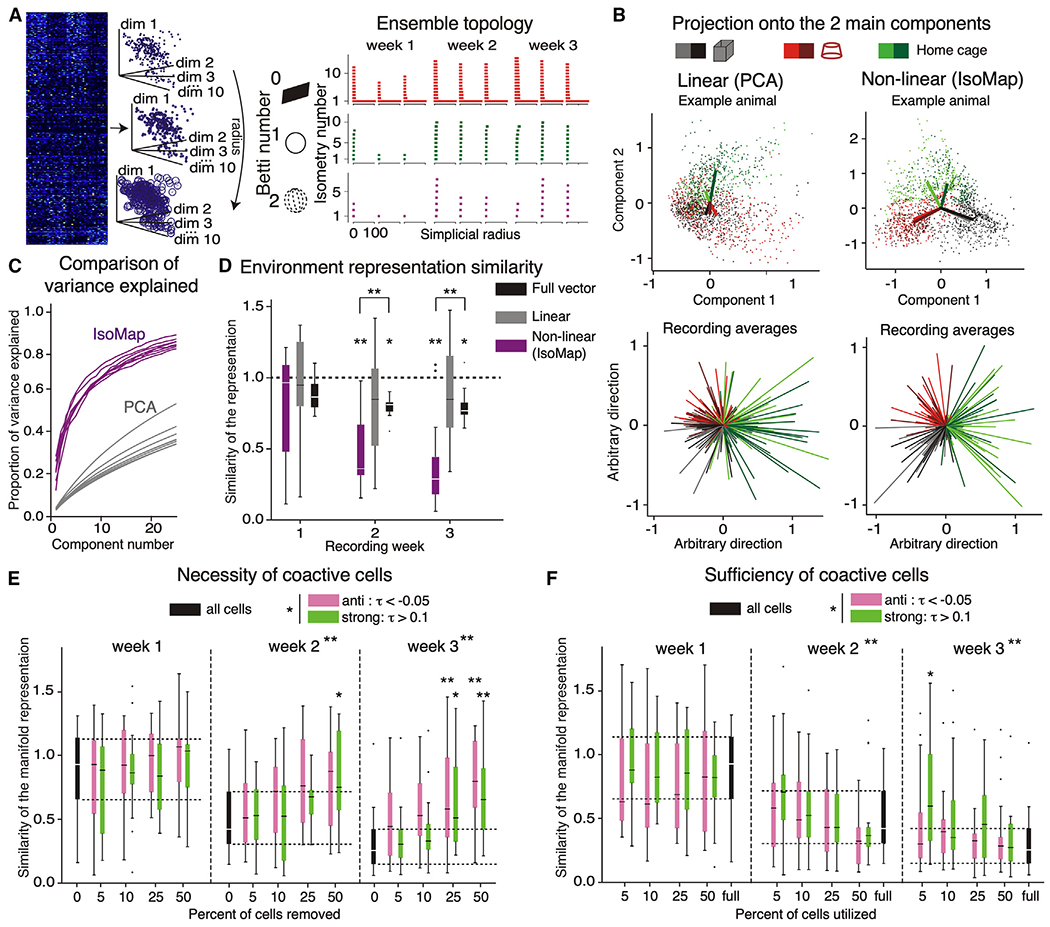
Non-linear embedding distinguishes environments reliant on anti-coactive cell pairs (A) Left: schematic of determining Betti numbers. Right: example Betti barcode; only Betti number 0 isometry persists. (B) Activity vectors projected onto a 2D subspace using PCA (linear) or IsoMap (non-linear) dimensionality reduction. In each recording, the average 2D vector is computed as shown (top) example with projections of individual 1-s activity vectors (points), and (bottom) dataset. Repetitions of the same environments on the same day are given distinct color shades. (C) Explained variance versus PCA and IsoMap dimensions. (D) Similarity ratio of the difference between average projections from same versus different environments using IsoMap, PCA, or the full activity vector (method: F_2,39_ = 10.38, p = 10^−4^, $\eta^{2}=0.19$; week: F_2,38_ = 6.67, p = 0.003, $\eta^{2}=0.76$; interaction: F_4,44.58_ = 2.96, p = 0.03; post hoc Dunnett’s tests: IsoMap < full vector, **p ≤ 0.046; post hoc Dunnett’s tests: week 1 > weeks 2, 3; *p < 0.03, **p < 0.01). (E and F) Similarity ratios after (E, necessity) removing or (F, sufficiency) using cell subsets according to their contribution to coactivity levels. Necessity (coactivity level: F_1,99_ = 6.06, p = 0.016, $\eta^{2}=0.012$; percentage: F_1,99_ = 17.86, p = 10^−5^, $\eta^{2}=0.078$; week: F_2,98_ = 22.08, p = 10^−8^, $\eta^{2}=0.13$; no interaction, all p > 0.18; post-hoc: anti > strong t_380.93_ = 2.11, p = 0.036; post hoc Dunnett’s tests: week 1 > weeks 2, 3; **p ≤ 10^−7^; post hoc t tests vs. all cells, *p < 0.05, **p < 0.01). Sufficiency (coactivity level: F_1,99_ = 5.27, p = 0.24, $\eta^{2}=0.014$; percentage: F_1,99_ = 4.71, p = 0.032, $\eta^{2}=0.025$; week: F_2,98_ = 13.00, p = 10^−6^, $\eta^{2}=0.17$; week × percentage × coactivity levels: F_2,98_ = 3.10, p = 0.049, $\eta^{2}=0.0055$; no other significant interaction, all p > 0.12; post hoc: anti < strong t_380.40_ = 2.33, p = 0.020; post hoc Dunnett’s test: week 1 > weeks 2, 3; **p < 0.01; post hoc t tests vs. all cells, *p < 0.05). For all boxplots, the height is determined by the interquartile range and the median is indicated with a line. The whiskers extend to the furthest data point up to 1.5 times the interquartile range past the boxplot limit. Data points past the whisker limits are either plotted individually or omitted when too large for better visual representation. Statistically significant differences are indicated on the plots. A statistically significant difference is indicated by one asterisk (p < 0.05 or the corresponding corrected value) and two asterisks (p < 0.01 or the corresponding corrected value).

The topology of 1-s CA1 ensemble activity patterns has one component and no holes (Figure 6A),^87^ which could approximate a 2D surface (Figure 6A; Video S1). IsoMap is non-linear (Video S2),^88^ so we compared it with principal-component analysis (PCA). We also used the replica mean field theory of manifolds to compute three geometric measures—capacity, extent, and dimensionality—without any dimensionality assumptions.^89^ IsoMap distinguishes the 1-s ensemble activity vectors recorded in the environments (Figure 6B, right) better than PCA (Figure 6B, left). The first 10 IsoMap and PCA dimensions explain ~70% and ~20% of the variance, respectively (Figure 6C). To quantify environment discrimination, we calculated the average projection onto the two main dimensions of IsoMap or PCA (Figure 6D). The difference between projections from the same environments was conservatively normalized by the difference between projections from different environments (Figure S9). This normalized vector difference, a discrimination ratio, decreases from 1 (less distinct) to ~0.3 (more distinct) with experience (Figure 6D). This contrasts with the modest decrease (>0.8) using PCA. The discrimination ratio only decreased modestly when it was computed on the raw activity vectors without any dimensionality reduction (Figure 6D). Finally, to elucidate whether the contribution of coactivity is necessary and sufficient for discriminating the environments, we recomputed the activity vector IsoMap projections after systematically removing or including only the top 5%, 10%, 25%, and 50% of cells that participated most in the coactive and anti-coactive cell pairs. Removing the most coactive or anti-coactive cells selectively degraded the IsoMap discrimination (Figure 6E), and including only the most coactive and anti-coactive cells was uniquely sufficient for the discrimination (Figure 6F). These effects were stronger for the anti-coactive cells.

### Registering and reregistering manifold geometry to environments

**Figure 7.**
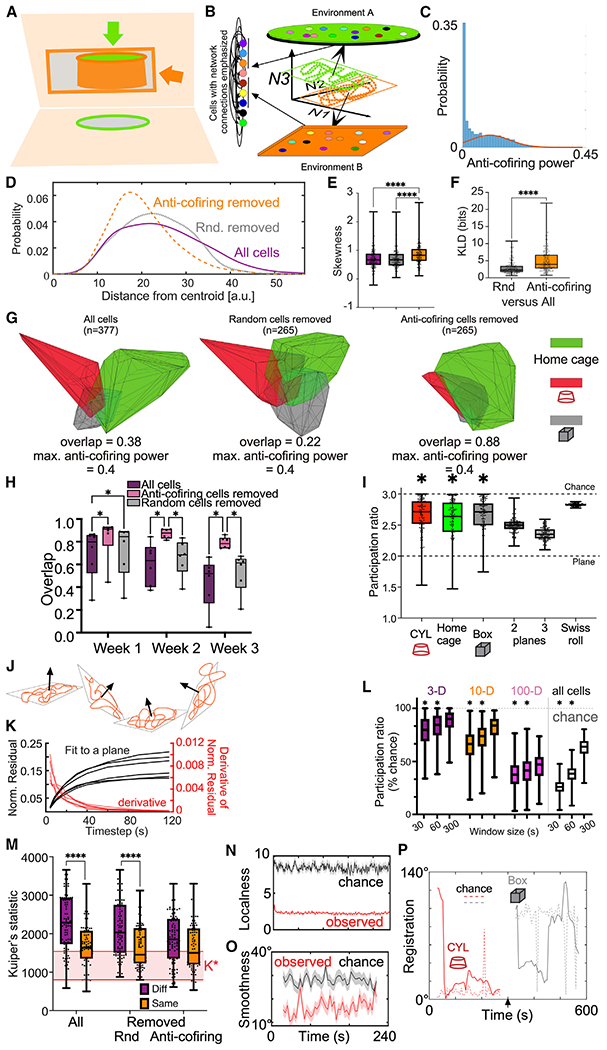
Reregistration, reconceptualizing how the hippocampus represents multiple environments (A) Distinct projections of an invariant object (a solid cylinder) can generate distinct representations (circle or rectangle). (B) The reregistration hypothesis illustrated. (C) Distribution (blue) of the anti-cofiring power computed for all recorded cells, with a Gaussian fit of the tail (orange). (D) A day 7 recording example histogram of 300 1-s activity vector distances from the centroid after projecting a 377-cell (all) ensemble into a 60D IsoMap subspace. The 112 (30%) most anti-cofiring or random cells were removed, and the remaining 265-cell ensembles were projected into a 60D IsoMap subspace. (E and F) Summary quantification of (E) skewness and (F) KLD of the 3 histograms from each recording (n = 200) confirm that anti-cofiring cells are distinctive and significantly distant from the centroid. (G) Visualization as a volume after projecting the activity vectors from (D) into a 3D IsoMap subspace. The component coordinate system was determined from two 5-min recordings in the home cage, box, and cylinder, with the home cage activity at the origin. Comparison of volumes occupied by activity vectors for all cells (left), a random subset (center), or after removing the most anti-cofiring cells (right). (H) Summary of all ensembles recorded across 3 weeks (two-way ensemble × week ANOVA, ensemble: F_2,45_ = 14.04, p = 10^−5^; week: F_2,45_ = 7.33, p = 0.002; interaction F_4,45_ = 0.32, p = 0.9; Friedman statistic = 30.79, anti-cofiring removed > all cells = randomly removed). (I) Participation ratios (PRs) to compare dimensionality of projections into 3D IsoMap subspaces (Kruskal-Wallis statistic = 138.2, p = 0). (J) Hypothesis that ensemble activity in the 3D IsoMap subspace is organized along planar trajectories that episodically shift orientation. (K) Residuals and derivative from best fit planes for each mouse as a function of the data time step used to estimate that the duration ensemble activity is constrained to a plane in the 3D IsoMap subspace. (L) PR data dimensionality estimates by projecting different windows of data into IsoMap subspaces of different dimensionality. (M) Kuiper’s statistics comparing the set of angles of the best fit planes in the 3D IsoMap subspace for each of the daily recordings in the same or different box and cylinder environments. (N–P) Summary measures of the (N) localness and (O) smoothness time series of the 1-s ensemble activity trajectories in the 3D subspace compared with chance and (P) an example time series (4-s time step) of the angular registrations of the best fit plane to 60 s of data along the ensemble trajectory through the 3D IsoMap subspace. For all boxplots, the height is determined by the interquartile range and the median is indicated with a line. The whiskers extend to the furthest data point up to 1.5 times the interquartile range past the boxplot limit. Data points past the whisker limits are either plotted individually or omitted when too large for better visual representation. Statistically significant differences are indicated on the plots. A statistically significant effect of the factor on the x-axis is indicated as a line above the graph. with asterisk(s). One asterisk indicates p < 0.05 (or the corresponding corrected value) and four asterisks indicate p < 0.0001 (or the corresponding corrected value).

These findings motivate a “reregistration” hypothesis where CA1 ensemble coactivity can discriminatively represent distinct environments through a relatively conserved population activity pattern that is constrained on a non-linear surface that is at once conserved across environments and distinctively registered to each environment (Figure 1B) by experience-dependent anti-coactivity (Figure 6; Figure S8). The hypothesis asserts that environment-specific differences in cofiring (Figure 3; Figure S8), are largely due to the anti-cofiring cells, which are responsible for the differences between environment-specific manifolds of neuronal activity, and the fact that the topology of the environment-specific manifolds is invariant (Figure 7B). The cofiring power distribution has a long anti-cofiring tail (Figure 7C). Both cells of an anti-cofiring pair tend to be active, rather than one cell active and the other silent (Figure S10A).

Are anti-coactive cells responsible for environment specificity? Each set of 1-s activity vectors was projected into a 60D IsoMap subspace, and each Euclidean distance from its 60D centroid was examined. The example of a 377-cell ensemble recorded on day 7 illustrates that the distribution has a long tail of “outlier” vectors that are far from the centroid (Figure 7D). We tested whether the most anti-coactive cells disproportionately contribute to the outliers by removing either the 30% (112) most anti-coactive cells or an equivalent random sample and then recomputed the histogram. Removing the anti-coactive subset disproportionately reduced the outliers, strongly shifting the distribution leftward, more than the random removal. This impression was quantified by skewness of the three distributions for each recording across the mice (Figure 7E; Friedman statistic = 69.12, p < 0.0001; 3 distributions, each with 200 samples; anti-coactive cells removed > all cells = random cells removed, p < 0.0001). Removing anti-coactive cells increased the Kullback-Liebler divergence (KLD) from the histogram of the full ensemble far more than removing random cells (Figure 7F; Wilcoxon W = 18,260, p < 0.0001). These findings suggest that anti-coactive cells cause distinctive activity vectors, which was confirmed using the replica mean field theory of manifolds to evaluate the relative change of dimensionality and extent measures after removing anti-cofiring cells versus random cells. The relative change in the extent of the manifold (5.83% ± 0.76%) is larger than the increase in dimensionality (3.27% ± 0.56%; paired t test t_49_ = 6.14, p = 10^−7^), hinting that anti-coactive cells are closely linked to the variability of the anchor points on the margin of the manifolds, thus changing the extent of these manifolds (Figure S10B).

To visualize and test the possibility that anti-cofiring cells are responsible for the distinction between the environmental representations, we projected the activity vectors from the pair of cylinder and box recordings on a particular day into a 3D IsoMap subspace determined by concatenating the home cage, box, and cylinder recordings of 1-s activity vectors, with the home cage activity centered at the origin. The volumes occupied by the 600 (2 × 5 min) 1-s ensemble activity vectors from the 377-cell ensemble recorded on day 5 show that the overlap of the cylinder and box activity is modest (0.38; Figure 7D), consistent with ensemble activity being sufficiently distinctive to discriminate between the two environments (Figure 6). Removing a random 30% of cells did not increase the overlap (0.22), whereas removing the 30% most anti-coactive cells reduced the outliers, did not remove enough cells to change the maximum anti-cofiring power, and increased the cylinder and box overlap (0.88). The ability of the anti-cofiring cells to distinguish the cylinder and box ensembles developed with experience; during the first week, the cylinder and box activity vectors occupied a largely overlapping subspace, but with experience, from week 2, they became more distinct (Figure 7H). Removing the anti-cofiring cells reduced the maximum anti-cofiring power from 0.20 ± 0.013 to 0.15 ± 0.013 and reduced the distinction between the two environments, but not when an equivalent random subset of cells was removed (maximum anti-cofiring power 0.19 ± 0.013). The relatively reduced distinctiveness of removing anti-cofiring cells is also observed as decreased capacity (−5.13% ± 0.74%; Figure S10B) of the population activity, also as predicted by the reregistration hypothesis.

### Multistable manifold representations of environments

Projecting activity vectors into the volume of the first three IsoMap components hinted that they may organize on 2D surfaces (Videos S1 and S3), which is corroborated by computing participation ratios (PRs) to estimate dimensionality of the 3D projections (PR = 2.65 ± 0.30, n = 258).^90,91^ PRs are significantly lower than chance (PR = 3) but indistinguishable from a Swiss roll or 2D planes that change orientation within the 3D space (Figure 7I).

Entorhinal and hippocampal spatial representations are multistable, spontaneously changing registrations to the environment on the timescale of ~10 s during navigation.^29,38,48,69,71,79,92^ If CA1 population activity intermittently registers to distinct environmental features, then it would change the orientation of a 2D surface within the 3D IsoMap space (Figure 7J).

Consistent with the multistable conjecture, 30–60 s of activity vectors are well fit to a plane in the 3D IsoMap subspace (Figure 7K). Video S3 shows that the ensemble activity takes nonrandom, smooth trajectories through a 2D subspace that intermittently changes orientation. PRs computed on 30-, 60-, and 300-s time windows (Figure S10C) indicate that the dimensionality is significantly lower for the shorter time windows (Figure 7L), suggesting that the hypothesized multistability occurs on a ~60-s timescale.

Kuiper’s statistic measured whether the set of 60-s planar angles differed for daily recordings in the same and different box and cylinder environments. The angles are more similar in the same than in different environments (Figure 7M), indicating that manifold orientation in the subspace can distinguish the two environments. This discrimination is lost when anti-coactive but not random cells are removed (two-way environment × ensemble ANOVA, environment: F_1,534_ = 56.87, p = 10^−13^, ensemble: F_2,534_ = 6.77, p = 0.001; interaction F_2,534_ = 5.06, p = 0.007; post hoc Tukey: different environments: anti-coactive removed < all cells = randomly removed p < 0.02; same environments: anti-coactive removed = all cells = randomly removed, p > 0.98). The localness (Figure 7N; 2.26 ± 0.01 vs. chance: 8.44 ± 0.04, Wilcoxon W = 8.77 × 10^8^, p < 10^−4^) and smoothness (Figure 7O; 16.5° ± 0.4° vs. chance: 28.0° ± 0.39°, Wilcoxon W = 1.68 × 10^7^, p < 10^−4^) of the ensemble trajectories in the IsoMap subspace are better than chance and together indicate that ensemble activity projected into the three best components of the IsoMap subspace is largely constrained to a 2D plane for several tens of seconds, and then, within 1–2 s, the plane reorients in the IsoMap subspace (Figure 7P).

Analogous to firing rate maps, the spatial distribution of each of the three principal IsoMap components of the ensemble activity vector projections were computed to investigate the possibility of time-averaged spatial tuning (Figure S11A). No obvious patterns were recognized, but they were also not random because, within week 2 recordings (day 4, 5, or 6), the patterns across the same environments were significantly correlated for 4 of 6 mice (10 of 36 significant correlations) and across the different environments for 5 of 6 mice (7 of 72 significant correlations). Finally, we examined the activity vectors around the times of the reorientations but did not recognize any relationships (r < 0.14, p > 0.2; Figure S11B).

## DISCUSSION

### The roles of feature tuning, coactivity, recurrence, and stability in the neural space code

Instead of single-cell properties, our analyses focused on cell-pair population coactivity that approximates higher-order correlated activity that can define informative neural population activity patterns (Figure S3).^44,46,78^ We imaged the activity and positions of hundreds of cells over weeks (Figure 2), an ability that was not available when the importance of place fields and remapping was established.

We replicate that single cells change place fields between environments and reinstate the fields upon imminent return to an environment (Figures 2F and 2H). We also document that place cell activity is temporally and spatially unreliable across days, even in familiar environments (Figures 2F and 2J).^16,35^ We identified recurrence of sub-second coactivity relationships among CA1 cells. The recurrence is modest (Figure 3C) but sufficient for differentially representing environments as well as locations (Figure 4B; Figure S5). Such variability seems at odds with expectations of stability for a memory and navigation system, but variable, distributed activity can organize within the stability of a manifold representation that explicitly does not depend on the reliability of either single-cell or population activity the way that conventional place field-based and activity vector-based spatial codes have been conceived (Figures 6 and 7).^43,50,93^

Cofiring analyses are neurocentric, based on the activity of other neurons, rather than external variables like places, and are effective for elucidating hippocampus^94–97^ and MEC^45,48,54,98^ function. The present findings offer an alternative concept for how hippocampal activity can be persistently informative about the environment without firing field stability (Figure 2) or even population activity stability (Figure 3; Video S2). A connectivity weight matrix, the hypothesized synapsemble,^99,100^ can transform the non-linear low-dimensional projections of variable neuronal activity (Figures 6 and 7) into a steady readout (e.g., environment identity and location) so long as the connectivity matrix is invariant to shifts in the manifold axes. In this case, the manifold would subserve memory persistence despite instability of neural activity, as demonstrated using artificial neural networks.^60^

### Limitations of the study

The timescale of analysis was restricted to 1 s because processes faster than ~300 ms are precluded by the slower timescale of GCaMP6f transients. Nonetheless, 1-s is the behavioral timescale of CA1 synaptic plasticity mechanisms^101,102^ and sufficient for computing the session-long cofiring relationships because the statistics of 25- and 40-ms cofiring relationships are well captured by 1- to 5-s cofiring relationships.^48,103^ We restricted analyses to anatomically separated cell pairs to avoid spuriously high activity correlations, preventing investigation of fine-scale topographical organization.^104,105^ Place field stability was variable, with some cells unstable and others highly stable. Stability increased after the first 3 days (30 min total) of exposure (Figures 2F and 2G), reaching a magnitude typical of mouse place cells recorded with tetrodes during purposeful behavior.^64,106^ Network consistency also increases with experience and with coactivity-based discrimination of environments and locations. This highlights the importance of coactivity (Figure 4; Figure S5), consistent with modeling results (Figure 1; Figure S2), and reports that CA1 place field plasticity relies more on population cofiring statistics than on the activity of individual cell pairs.^101^ Coactivity relationships were scale free (Figure S4) and dominated by the minority subpopulation of anti-coactive cells for discriminating environments (Figures 4–7), resembling flocks and their interactions with external features^84–86^ and Pareto’s principle.^107^ SVM decoding measured globally optimal contributions of each cell or cell pair to distinguishing environments (Figures 4 and 5) and locations (Figure S6). We used the IsoMap algorithm to discover that somewhat variable ensemble activity was constrained to lower-dimensional subspaces by distinct environment-specific patterns of coactivity (Figures 6 and 7). We analyzed ensemble activity within 60D IsoMap subspaces that account for almost all of the variance (Figure 6C) as well as assuming a temporally local 2-D organization within a 3D IsoMap subspace to evaluate the multistability conjecture without assuming knowledge of the true geometry or that the true dimensionality is 2D, as argued by others.^108^ Despite such simplifying assumptions, the findings are robust; they hold when we applied the replica theory of mean field manifolds that measures rather than assumes the network geometry without dimensionality reduction.^89^ These findings constrain possible neural mechanisms for reading out the information represented in coactivity and point to synapsemble properties^99,100^ that, in artificial neural networks, have been demonstrated to be effective mechanisms for transforming variable input activities to a reliable readout.^60^

### Reregistration instead of remapping to represent spaces and memory

CA1 ensemble activity is only modestly different across environments (Figures 3 and 7) and within an environment exhibits multi-stable dynamics (Figure 7) that resemble remapping expectations.^7,30,38,40^ The data are consistent with the alternative concept of reregistration. Reregistration assumes that CA1 activity is neurocentric, internally organized in a relatively invariant manner, such as on a low-dimensional manifold (Figures 7A and 7B). Instead of external stimuli causing rearrangements of neuronal activity, the neurocentric activity maintains but variably registers to external stimuli.^109^ This process may resemble fitting a model to observations (Figure 7B). Cells that are most strongly influenced by currently salient stimuli are most likely to change their firing, and because of the scale-free network correlations (Figure S4D), they influence and are influenced by the activity of all other cells. In flocks, this allows the activity of most individuals to be maintained locally while the entire flock changes globally without changing its dynamics.^84–86^ The result of reregistration is that only a minority of cells change their activity distinctly from the activity of the other cells in the network, and these, we speculate, are anti-cofiring cells (Figures 4–7). While we cannot exclude the possibility that anti-cofiring cells signal environment-specific behavioral or brain-state variables (for instance, “happy” and “sad”) that are unique in each environment, we did everything that is standardly done to homogenize behavior and brain states across the environments to avoid such confounds. CA1 activity retains its low-dimensional organization but is registered uniquely to the environment in a manner that persists, especially, we speculate, when synaptic weights are preferentially adjusted between the anti-cofiring cells and their environment-associated inputs. Because place cell firing is fundamentally multimodal, only appearing unimodal in small environments,^8,9^ the ~25% of CA1 cells that express firing fields change firing field locations with reregistration, although the neurocentric organization of activity changes much less, as confirmed by simulations (Figure 1; Figure S2). Reconceptualizing remapping as reregistering identifies the importance of the anti-cofiring cell subset not only as crucial for discriminating environments (Figures 4–7) but also for orienting the relatively invariant manifold activity patterns within the neuronal activity subspace (Figure 7).

We struggled to establish a firm relationship between changes in firing fields and memory,^24,110,111^ but, in line with the original conceptualization of remapping as a temporal reorganization of discharge,^4,112^ memory correlates have been identified in changed cofiring relationships between E as well as I cells in sleep and active behavior.^23,69,113,114^ Such changes in coactivity are consistent with synaptic plasticity studies that demonstrate balance,^115^ bidirectionality,^101^ and involvement of E and I cells.^67,68,71,116,117^ We find that the strongly coactive and anti-coactive cell pairs are particularly discriminative at the 1-s timescale, and anti-coactive cells are more discriminative and more important for neurocentrically orienting the activity on a manifold (Figures 6 and 7; Figure S8).^118^ This is consistent with the increase in interneuron-principal cell cofiring at moments of memory discrimination and recollection.^23,69^ Using PTI, we analyzed activity fluctuations around place tuning,^119^ removing the place signal from the time series. We did this to compute cell-pair coactivity beyond cofiring because of place tuning and found that it improved the ability to discriminate environments (Figure 4; Figure S1), hinting at independence of the place and environment codes. We focused on the role of cofiring, which has been far less studied than the role of place fields, and both features of neural activity likely contribute to neural mechanisms of spatial cognition. There are precedents of parallel codes for distinct spatial variables.^33,41,42,62,120–122^ The coexistence of place field and coactivity codes for different features of space in hippocampal neural activity predicts that the explicit memory-related information in CA1 activity that has evaded the field’s efforts would manifest in short-timescale coactivity, although, in our view, the manifold organization of ensemble coactivity relationships is more likely the proper description of the hippocampal spatial code. Although we identified a correspondence between patterns of neural coactivity and the ability to discriminate environments, we have not determined what is actually represented in neural activity, nor whether this correspondence causes the subject to understand its environments as distinct, or anything else for that matter. Nonetheless, our findings demonstrate the value of a conceptual shift toward a serious consideration of such vexing problems from the vantage point of the collective and inherently temporally structured neurocentric behavior of neural activity.^58,123^

## STAR★METHODS

### RESOURCE AVAILABILITY

#### Lead contact

Further information and requests for resources should be directed to the Lead Contact, Andre A. Fenton (afenton@nyu.edu).

#### Materials availability

This study did not generate new unique materials.

#### Data and code availability

- Calcium imaging and behavior data have been deposited at a Harvard Dataverse repository and are publicly available as of the date of publication. The DOI is listed in the key resources table.
- All original code has been deposited at a Harvard Dataverse repository and is publicly available as of the date of publication. The DOI is listed in the key resources table.
- Any additional information required to reanalyze the data reported in this paper is available from the lead contact upon request.

### EXPERIMENTAL MODEL AND STUDY PARTICIPANT DETAILS

#### Animals

A total of 41 wild-type adult male and female C57BL/6J mice were used for the study, obtained from Jackson Laboratory (strain #000664). Six male mice contributed data. Mice were 3–6 mo at the time of the experiments. The mice were maintained on a 12:12 light:dark cycle and single housed after surgery. All work with mice was approved by the New York University Animal Welfare Committee (UAWC) under Protocol ID: 17–1486.

### METHOD DETAILS

#### Virus injection and lens implant

To virally infect principal cells to express the fluorescent calcium indicator GCaMP6f, adult C57BL/6J mice (n = 41) were anesthetized with Nembutal (i.p. 50 mg/kg), 1 h after receiving dexamethasone (s.c. 0.1 mg/kg). They were mounted in a stereotaxic frame (Kopf, Tujunga, CA) and through a small craniotomy, they were injected into the right CA1 subfield (AP: 2.1mm, DV: 1.65 mm, ML: 2 mm) at a rate of 4 nL/s with 0.5 μL AAV1.Syn.GCaMP6f.WPRE.SV40 (titer: 4.65 × 1013 GC/mL; Penn Vector Core). The injection pipette (Nanoject III, Drummond) was left in place for 5 min before it was slowly withdrawn. Thirty minutes later, a larger craniotomy was performed with a 1.8-mm diameter trephine drill and the overlying cortex was removed by suction. A gradient-index ‘GRIN’ lens (Edmund Optics, 1.8 mm diameter, 0.25 pitch, 670 nm, uncoated) was implanted, fixed with cyanoacrylate, and protected by Kwik-Sil (WPI, Sarasota, FL). The skull was covered with dental cement (Grip Cement, Dentsply, Long Island City, NY). The mice received one slow-release buprenorphine injection (s.c. 0.5 mg/kg), dexamethasone (s.c. 0.1 mg/kg) for 6 days, and amoxicillin in water gel for a week. Three to eight weeks later, once a good field of view was visible through the GRIN lens, the baseplate for a UCLA miniscope was implanted and fixed to the skull with dental cement www.miniscope.org.^124^;

#### Behavior

After recovery from surgery, animals were handled for a couple minutes 1–3 times a week until the baseplate was implanted. The animals were then habituated to wearing the miniscope in their home cage, first in the animal holding room and then in the experimental room. Once the animal was comfortable wearing the miniscope, we started the behavior experiment and recording.

The mice were exposed to two environments, a 32 cm-diameter circle with transparent walls and a 28.5 cm-side square with opaque black walls and distinctive orienting pattern on 3 of the walls. The surface area of the two enclosures were similar, within 2% of 800 cm^2^, but the floor of each environment was also distinctive; the circle’s floor was made of red plastic while the rectangle’s was of black metal. Orienting cues were also present in the room, and directly visible from the circular enclosure; the door and the animal transport cart were visible, and two salient cues were present on opposite walls of the room.

The mice explore each environment twice, for 5 min each time, in an interleaved fashion. The animal was placed in its home cage between trials for a couple minutes to allow us to change environments. Windows-based software (Tracker 2.36, Bio-Signal Group Corp., Acton, MA) determined the mouse’s location in every 30-Hz video frame from an overhead digital video camera.

This protocol was repeated for three consecutive days, every week of three consecutive weeks. For two animals, this was repeated in periods of 5 days (instead of 7). For all animals, we started calcium recording on the day the animal first explored the two environments. We aimed to record the entirety of the 5-min visits to the circle and square environments and 5-min sessions in the animal’s home cage were also recorded each day before and after the 4 sessions in the two experimental environments.

#### Calcium recording and signal extraction

Neural activity data from 6 of the 41 mice met the quality analysis requirements described below and were analyzed to address the central question. When recording, the UCLA miniscope was attached to the baseplate, and fluorescent images from CA1 were recorded through the GRIN lens using the UCLA miniscope data acquisition hardware and software (www.miniscope.org). Thirty images were collected each second and analyzed offline. Before analysis, we screened the average calcium level of each recording and removed any day or week where large and abrupt changes to the mean fluorescence were observed. The images from each recording were aligned using the NoRMCorre algorithm.^125^ The alignment was done separately for each recording but all recording alignments from a single mouse used the template generated by the alignment of the first video recorded that day. Aligned videos were then subsampled to 10 Hz by averaging and the recordings from each day were concatenated into a single file. Action potential activity was then estimated separately for each day from the ΔF/F GCaMP6f signal using the CNMF-E algorithm,^75,76^ which simultaneously separates the cells’ fluorescence and deconvolves the calcium transients to infer spiking activity. Accordingly, we refer to ‘activity’ rather than ‘firing’, especially because calcium transients are likely to reflect bursts of action potentials rather than single action potentials.^126^ The spatial footprint of the cells were seeded using a peak-to-noise ratio determined manually for each recording session. Units with high spatial overlap (>0.65) and high temporal correlation (>0.4) were merged and the CNMF-E algorithm was updated until no pair of units meet the criteria. Ensembles were evaluated manually for quality control to reduce the likelihood that artifacts were identified as cells. Furthermore, recordings were examined for evidence of photobleaching and minutes scale downward or upward trends in activity by regression analysis of the activity timeseries of individual cells for every one of the ≤54 (cylinder, box, home cage) recordings (6/day X 3 days × 3 weeks). Only recordings with no significant linear trends and without abrupt decreases in activity levels were studied.

To evaluate the integrity of the neuron ensembles we recorded, we used two metrics: temporal independence and spatial dependence. Temporal independence evaluated for each cell the average cell-pair correlation with all cells whose center is within 20-μm of the soma of the cell. Spatial dependance measured the proximity and overlap with nearby cells. For each cell, the CNMF-E algorithm computes a spatial footprint. The spatial dependence is the proportion of the summed footprints within a 20-μm radius area around the soma that is not attributable to the cell itself. These metrics confirmed to the best of our abilities, that no corrupted unit or ensembles were included in the dataset. Ensembles ranged in size from 39 to 588 isolated units, with half larger than 260 cells and less than 10% smaller than 100 cells.

#### Alignment and cell matching

To match cells across days, every pair of recording days was first aligned to each other using the template used for the within-day alignment. This alignment was done using a non-rigid optical flow algorithm,^127^ calcOpticalFlowFarneback function in the cv2 python library) whose parameters (window size, flow levels, number of iterations) were optimized for each animal. Overlapping spatial footprints were then matched conservatively (matches were performed using the Hungarian algorithm^128^ with a maximum distance considered or cost of 0.7). Alignment and cell-matching algorithms were applied as implemented in the register_ROIs function of the CalmAn project.^129^ Day-to-day alignments were verified by eye and for each alignment an f1-score was computed as the ratio of the number of cells aligned to the total number of cells. Alignments with an f1-score below 0.3 were discarded.

### QUANTIFICATION AND STATISTICAL ANALYSIS

#### Place cell identification

We computed cell-specific spatial activity maps from each cell’s activity, extracted at 10 Hz, and the animal’s location, subsampled to 10 Hz, by computing the average rate of the cell in each ~2.5 × 2.5 cm bin. Linearized spatial activity maps were computed by changing the Cartesian coordinates to circular coordinates, with the origin at the center of the environment. We then computed the average rate of the cell in each 12° bin with the distance from the origin ignored.

To identify place cells, we used two metrics: Spatial coherence and information content.^130,131^ Spatial coherence was defined for each cell as the correlation between the average rate at each location and the average of the average rate at the locations neighboring this location (up to 8). Information content was defined for each cell as:

$$InfoContent=\sum_{positions\;x}p\left(x\right)\frac{r_{x}}{R}\log_{2}\frac{r_{x}}{R}$$

where p(x) is the probability of the animal being at postion *x*, *r_x_* the average rate at position *x* and *R* the average rate of the cell.

For each cell we computed both metrics as well as a distribution of randomized values with shuffled activity. The metric *val* was considered significant if

$$\frac{val-val_{random}}{stdev\left(val_{random}\right)}>1.96$$

with $val_{random}$ being the distribution of randomized values.

We classified a cell as a place cell only when both metrics were significant.

#### Place field similarity

The stability of a cell’s spatial tuning was assessed by comparing the spatial activity map in a pair of recordings. The Pearson correlation was computed between the pairs of activity rates at corresponding pixels in the activity rate maps. The average correlation (r) is reported for summary statistics but for statistical comparisons $z=\frac{1}{2}ln\frac{\left(1+r\right)}{\left(1-r\right)}$, Fisher’s transform was used.

#### Cell-pair correlations

Kendall tau (τ) correlations were computed using 1-s time bins because this measure of association is robust to time series with a low range of values and many ties (e.g., 0 and 1). We evaluated cell-pair correlations as a function of the distance between neurons and found that neurons within 25μm of each other were, on average, more strongly correlated. Since our recording method does not allow us to determine whether this is physiological or an artifact, we decided to exclude all such pairs. Therefore, for all analyses using cell-pair correlations, we excluded cells pairs that were not spatially separated (distance <30μm). We also excluded cells with very low activity (active less than 2% of 1-s time bins) as Kendall correlations computed on such time series have little meaning.

#### Position-tuning independent (PTI) rate

From the rate maps (average rate at each position), we computed the expected rate as the average rate at the animal’s location/the sampling rate (here 100 ms). The expected rate is then binned into 1-s bins and subtracted from the 1-s binned observed rate.

#### Support vector machine (SVM) decoding

SVM analysis was performed on concatenated 1-s binned activity time series from both environments using the sklearn python library. Training and decoding are performed 100 times on each dataset using different randomly separated training set (2/3 samples) and a testing set (1/3 samples). Decoding accuracy and features (cells or cell pairs) weights are then averaged across the 100 repetitions. This ensured that the results were not sensitive to random variations in the dataset split. Decoding accuracy is computed as the number of right decisions in the testing set. When decoding and comparing weights across day, no cross validation was used, and the entire reference day dataset was used to train the decoder. When decoding location, the SVM decoder was trained and evaluated using the 1-s activity vectors or coincidence vectors while the animal was in each of the two binned locations comprising the n(n-1)/2 pairs of bin locations, in each environment. Given n cells, the 1-s activity vector is the activity of each cell during the 1 s, whereas the 1-s coincidence vector is the coincidence of the n(n-1)/2 cell pairs scored as 0 if neither cell is active, 1 if only one cell is active and 2 if both cells are active in the 1 s. The location decoding score was computed as the average of correct choices between the n(n-1)/2 pairs of locations. When decoding environment, the SVM decoder was trained and evaluated in the 1-s activity vectors or coincidence vectors during each day (two recordings in the cylinder environment and two recordings in the square environment). The environment decoding score was computed as the average of correct choices, using 100-fold cross-validation. Because SVM decoding is sensitive to the number of elements (i.e., vector dimension) for comparisons of different types of input, the input data were downsampled to the lowest dimension vector that was being compared.

#### Network consistency

The Network consistency^23^ describes how well the momentary covariance in cell-pair activity fluctuations align to the overall activity correlation of the cell pair. Network consistency of an ensemble of time series is calculated as:

$$NetCo\left(t\right)\sum_{i,j}\tau_{i,j}*r_{i}\left(t\right)*r_{j}\left(t\right)$$

Where $\tau_{i,j}$ is the pairwise Kendall correlation between cell *i* and cell *j* and $r_{i}\left(t\right)$ the activity rate of cell *i* at time *t*. To compare network consistency values between animals we normalized it in each recording to the standard deviation of the distribution of values obtained when the identities of the cell pairs were shuffled 100 times. In the figure, we report the average network consistency for each recording.

#### Correlation participation

To quantify the role of cell-pair activity correlation in the SVM and IsoMap analyses, we evaluated for each cell their participation in correlations of different ranges of correlation t values. For each cell, we computed its participation in a range of t values as the number of times a cell participates in a pair with a t value within that range, divided by the total number of pairs in which the cell participates. In effect, it is the proportion of t values falling in a certain range amongst all cell pairs the cell participate in. In practice, this normalization has limited effect but allows us to remove any impact from the exclusion of some cell pairs.

#### Geometric properties of high-dimensional neural manifolds

As detailed in^89^ neural manifolds are defined by D+1 coordinates: one to describe the location of the manifold center, and D axes that define the manifold variability. In this work, we consider that each of the P manifolds is sampled M times along N different features. In this work, P manifolds correspond to P recordings of the animal in each environment (square and/or circle), and N features correspond to N neurons.

To characterize the separability among neural manifolds, we introduce the concept of classification capacity, $\alpha_{c}$. Classification capacity is understood as the maximum number of manifolds that can be linearly separated given random assignment of binary labels to manifolds, and mathematically as the ratio between the critical number of manifolds, *P_c_* and the number of features, N. Borrowing the concept of support vectors from the problem of linearly separable points, in manifold theory, the high dimensional separating hyperplane is defined by a linear combination of what we call manifold anchor points. Each manifold contributes with (at most) a single anchor point, which uniquely define the separating plane between manifolds. The identity of the anchor points depends on the location and orientation of the manifolds as well as the randomly assigned binary labels. As a result, for a given manifold, there is a statistical distribution of anchor points. From the distribution of anchor points, we can introduce two geometric properties of manifolds: the effective radius, *R_M_*, defined as the square root of the total variance of the anchor points, normalized by the average norms of the manifold centroids, and the effective dimension, *D_M_* which captures the dimensionality of the anchor points along the different manifold axes. We define the manifold total extent, a measure of the space occupied by the high dimensional neural manifold as the mean $R_{M}\sqrt{D_{M}}$. According to the replica mean field theory of manifolds, larger *R_M_*, *D_M_* and $R_{M}\sqrt{D_{M}}$ of the manifolds are linked to lower linear separability between these manifolds, indicating more overlap between them.

#### Betti numbers and dimensionality reduction

Principal Component Analysis (PCA) and IsoMap dimensionality reduction were performed each day separately on concatenated 1-s binned activity time series from both environments as well as the home cage, filtered with a Gaussian filter of 1-s standard deviation. Both transformations used the sklearn python library. IsoMap was performed using Euclidean distance and 5 neighbors.^88,132^ Betti barcodes were computed each day on a 10-dimension IsoMap projection using the Ripser algorithm^133^ in the scikit python library. To compute isometries of Betti number 2, the data were randomly subsampled to 700 data points (~40%) to allow for computational tractability.

#### Low-dimensional neural manifold analysis

We start by considering an N-dimensional neural manifold. The dimensionality of the neural manifold is evaluated using the participation ratio (PR), defined as:

$$PR=\frac{{\left[Tr\;C_{neuron}\right]}^{2}}{Tr{\left[C_{neuron}\right]}^{2}}$$

where $Tr\;C_{neuron}$ is the trace of the covariance matrix of a pattern of firing rates across M recorded neurons at some specified time window.^90^

To quantify the anti-cofiring properties of each cell, we introduce the anti-cofiring power, defined as the number of cell-pair correlations with τ<−0.05, normalized by the total number of correlations calculated for the cell (i.e., n-1).

Further, to describe the distribution of distances to the centroid in an N-dimensional manifold, we employ two different measures. First, we use the skewness as a measure of the asymmetry of the data around the sample mean, i.e., a measure of the tail-ness of the distribution, which contains the ‘outliers’, earlier shown to be most anti-cofiring cells, defined as:

$$skewness=\frac{\frac{1}{n}\sum {\left(x_{i}-\bar{x}\right)}^{3}}{{\left(\sqrt{\frac{1}{n}\sum {\left(x_{i}-\bar{x}\right)}^{2}}\right)}^{3}}$$

Second, to measure the similarity between distributions for different ensembles, we use Kullback-Liebler divergence (KLD). KLD is a measure of how one probability distribution *f*_1_ is different from a second, reference probability distribution *f*_2_. This measure can be interpreted as the expected excess surprise from using *f*_1_ as a model when the actual distribution is *f*_2_.

Then, we construct 2D neural manifolds using IsoMap projected neural activity into a three-dimensional subspace. The 2D manifolds are constructed using the in-built function *alphaShape* in MATLAB. The algorithm creates a 3D bounding volume using a triangulation algorithm. To identify the overlapping coefficient between two environments, we first identify the number of points from one environment manifold that belongs to the other environment manifold, using the in-built command *inShape*. We then compute the overlapping coefficient (overlap) by normalizing the number of occurrences from Environment 1 inside the 2D manifold of Environment 2, with the total number of points from Environment 1.

To explore the multi-stable planar manifold hypothesis, we compute the best fit plane to the 3D IsoMap projected point cloud data for each environment using an in-house MATLAB function. The algorithm fit 1-s activity vectors in a 1 min window that was advanced in 1-s increments. The algorithm fits a least square solution for the normal vector that defines the best fit plane. The algorithm minimizes the sum of the squared dot product of a trial normal vector with a vector passing through the centroid of the data. The output is a normal vector and the centroid of the 3D point cloud data.

To further quantify the distinction between environments, we compare the distribution of angles measured between the normal vectors of each pair of consecutive planar fits within a particular environment. These distributions were compared between repeated recordings of the same and different environments using Kuiper’s statistic, a rotation-invariant Kolmogorov-type test statistic.

Finally, to describe the trajectories inside the neural manifold, we introduce two quantities: localness, defined as the Euclidean distance between two temporally adjacent points, and smoothness, defined as the angle between the normal vectors of two temporally adjacent planar fits.

#### Statistics and visualization

All data were plotted using boxplots unless the distribution contains less than 10 data points, in which case individual data points are plotted. For all boxplots, the height is determined by the interquartile range and the median is indicated with a line. The whiskers extend to the furthest data point up to 1.5 times the interquartile range past the boxplot limit. Data points past the whisker limits are either plotted individually or omitted when too large for better visual representation.

Statistically significant differences are indicated on the plots. One asterisk indicates p < 0.05 (or the corresponding corrected value) and two asterisks indicate p < 0.01 (or corresponding corrected value). When indicated, a statistically significant effect of the factor week is indicated as a line above the graph with asterisk(s).

Within subject comparisons were performed, when possible, using paired statistics such as Student’s paired t test, and the corresponding non-parametric test, Wilcoxon test. Comparisons amongst multiple factors or amongst more than two groups were performed using ANOVA, with repeated measures (RM) when the effect of week was being evaluated. The Hotelling-Lawley correction was used when the data being compared by RM ANOVA violated the sphericity assumption as assessed by Mauchly’s Test of Sphericity. For analyses in which a particular percentage subset of the data was included or excluded, the factor of percentage was treated as a continuous variable with df = 1. Dunnett’s tests were used to evaluate post-hoc pairwise comparisons against a control, such as an initial week or control subset, when appropriate. Bonferroni-corrected multiple comparisons were used when only a subset of pairwise comparisons were of interest and when comparing multiple groups against a value or control group (if not present in the initial RM ANOVA). For comparison amongst multiple factors or amongst more than two groups where the data were not normally-distributed we performed Friedman’s statistical test and Kruskal Wallis test. All correlation data were Fisher z-transformed before statistical analysis using parametric methods. The test statistics and degrees of freedom are presented along with the p values and effect sizes for all comparisons, with only power of ten indicated when a value is below 0.001. A value of p < 0.05 was considered significant. The statistical analyses of data in a figure are presented in the corresponding figure legend.

#### Large environment model

Larger place field maps were created from the concatenations of the place field map of four different cells. Only cells that were categorized as place cells in at least one repetition of the two environments were considered. For each larger map, we obtained four firing field maps, one for each visit of the two environments. Cells from a single day (day 6) and from all six animals were used to create the 151- ‘composite’ cell ensemble. Results did not significantly change across days.

A random walk was then simulated across the larger environment but limited to different boundaries depending on the environment size evaluated. To simulate the random walk, we started at a random position within the allowed boundaries and randomly sampled a move every dt = 0.1s. The size of the large place field space was considered to be 24 × 24 space units. Position change was sampled randomly from [−1, 0, 1] and multiplied by a speed variable. The speed variable was initiated at 0.1 and, similarly to the position, was changed at each time step by a random sample from [−0.1, 0, 0.1], with 0.1 and 1 being the speed upper and lower bounds respectively. This corresponds to a maximum speed of 2.5–25 cm/s in real space.

Four different random walks were created for the two visits to the two environments and expected activity was created for each place map as described in the Position-tuning independent (PTI) rate method section. Environments were then decoded as described in the Support vector machine (SVM) decoding method section.

#### Spike-time dependent plasticity network model and analyses

The code implementing the spiking neural network model was custom written and has been made freely available at a Harvard Dataverse repository doi:10.7910/DVN/L0Z9AK.

#### Architecture

As illustrated in Figure 1B, the spiking neural network was organized as follows: The input to the network came from a layer of ${\text{n}}_{\text{input}}=1000$ neurons that were tuned to locations on the track. This population fed into a layer of ${\text{n}}_{\text{excit}}=500$ excitatory neurons with weights ${\text{W}}^{\text{InputE}}$. The connection between the input and excitatory populations were non-plastic. The excitatory population was both recurrently connected to itself $\left({\text{W}}^{\text{EE}}\right)$, and to an inhibitory population of ${\text{n}}_{\text{inhib}}=50$ neurons $\left({\text{W}}^{\text{EI}}\right)$. This inhibitory population is connected back to the excitatory layer $\left({\text{W}}^{\text{IE}}\right)$. The E→E, E→I, and I→E weights were all plastic.

At initialization, all values of ${\text{W}}^{\text{InputE}}$ were uniformly sampled from the interval $\left(0,W_{\max}^{\text{InputE}}\right)$. For ${\text{W}}^{\text{EE}}$, each weight either took a uniformly sampled value from the interval $\left(0,W_{\max}^{EE}\right)$, with probability $1-{\text{p}}_{\text{EE}}$, or was set to 0, with probability ${\text{p}}_{\text{EE}}$. The diagonal entries of ${\text{W}}^{\text{EE}}$ were all set and kept at 0. To ensure that the total amount of initial synaptic weight was conserved across the different weight populations, we set $W_{\max}^{EE}=W_{\max}^{\text{InputE}}/p_{EE}$. The same initialization procedure was repeated for ${\text{W}}^{\text{EI}}$ and ${\text{W}}^{\text{IE}}$ with $W_{max}^{EI}=W_{max}^{InputE}/p_{EI}$ and $W_{max}^{IE}=W_{max}^{InputE}/p_{IE}$.

#### Input layer

The input layer to the spiking neural network was made up of ${\text{n}}_{\text{input}}$ neurons, each tuned to at least one position on the linear track. The distribution of number of locations each input neuron was tuned to was binomial, with ${\text{p}}_{\text{input}}=0.20$. The centers of these tunings took a continuous value from [0, 10). The tuning to each center was taken to be Gaussian. That is, for neuron *i* with center, $c_{i}$, the tuning takes the form

$$T\left(\left|x-c_{i}\right|\right)=\exp\left(\frac{-{\left|x-c_{i}\right|}^{2}}{\sigma^{2}}\right)$$

where |⋅| is circular distance and *x* is a given location on the track. Neurons with n > 1 center take the sum of their tuning, which then takes the form

$$T_{i}\left(x\right)=\sum_{j=1}^{n}T\left(\left|x-c_{i}^{\left(j\right)}\right|\right)=\sum_{j=1}^{n}\exp\left(\frac{-{\left|x-c_{i}^{\left(j\right)}\right|}^{2}}{\sigma^{2}}\right)$$

The probability of input neuron *i* firing when at position *x* is proportional to the input with added noise such that, at any given time point, it is given by

$$P_{i}\left(x\right)=\left[T_{i}\left(x\right)+\alpha \cdot \epsilon_{pink}\right]\cdot \Delta t\cdot r_{PF}$$

where Δt is the time step length of the simulation, *r*_*PF*_ is the “in-field” firing rate, and $\epsilon_{\mathrm{pink}}$ is a noise term that is drawn from a pink noise distribution (https://github.com/cortex-lab/MATLAB-tools/blob/master/pinknoise.m) and is weighted by α. In the case of a negative noise term that is larger than $T_{i}\left(x\right)$, $P_{i}\left(x\right)$ was set to 0.

#### Neuron model

The neurons in the excitatory and inhibitory populations were modeled as simplified integrate-and-fire neurons. That is, the voltage of excitatory neuron *i* at time *t* is given by the weighted sum

$$V_{i}^{E}\left(t\right)=V_{i}^{E}\left(t-1\right)\cdot exp\;exp\left(-\Delta t\right)+f^{input}\cdot W_{i}^{InputE}+f^{E}\cdot W_{i}^{EE}-f^{I}\cdot W_{i}^{IE}+\alpha \cdot \epsilon_{pink}$$

where $f^{input}$ is the spikes of the input population and $W_{i}^{InputE}$ are the weights from the input layer to neuron *i* (and similarly for E and I being the excitatory and inhibitory populations, respectively). As in the case of the input layer, $\epsilon_{pink}$ is a noise term that is drawn from a pink noise distribution, which is weighted by α. When the voltage is greater than the threshold $T_{IF}$, the voltage is set to zero and $f_{i}^{E}$ is set to 1 for the next time step. We include an absolute refractory period, $\tau_{IF}$, where ${\text{V}}_{\text{i}}^{\text{E}}$ is kept at 0.

For inhibitory neuron *i*, the voltage is given by

$$V_{i}^{I}\left(t\right)=V_{i}^{I}\left(t-1\right)\cdot exp\;exp\left(-\Delta t\right)+f^{E}\cdot W_{i}^{EI}+\alpha \cdot \epsilon_{pink}.$$

#### STDP plasticity

All E→E, E→I, and I→E weights could be modified via spike-timing dependent plasticity (STDP) rules. In the experiments where one (or all) of the plasticity types were removed, then those weights were not subject to STDP, but were instead frozen.

The recurrent excitatory weights were modified at each time step via excitatory STDP that has the following form

$$\Delta W_{ij}^{EE}\left(t\right)=\eta \left(f_{i}^{E}\left(t\right)\cdot v_{j}^{E}\left(t\right)-f_{j}^{E}\left(t\right)\cdot v_{i}^{E}\left(t\right)\right),$$

$$v_{i}^{E}\left(t\right)=v_{i}^{E}\left(t-\Delta t\right)exp\left(-\Delta t\right)+\delta \left(1-f_{i}^{E}\left(t\right)\right),$$

where the convention that *i* is the post-synaptic neuron and *j* is the pre-synaptic neuron is followed. $f_{i}^{E}\left(t\right)$ is the spiking activity of the excitatory neuron *i* at time *t*, η is the learning rate (and maximum amount of change allowed at each time step), and δ is the Kronecker delta function. The second equation keeps track of the spiking history of each neuron.

The weights from the excitatory population to the inhibitory population were similarly modified

$$\Delta W_{ij}^{EI}\left(t\right)=\eta \left(f_{i}^{I}\left(t\right)\cdot v_{j}^{E}\left(t\right)-f_{j}^{E}\left(t\right)\cdot v_{i}^{I}\left(t\right)\right).$$

Finally, the weights that projected from the inhibitory population to the excitatory population were modified at each time step via inhibitory STDP that has the following form

$$\Delta W_{ij}^{IE}\left(t\right)=\eta \left(f_{i}^{E}\left(t\right)\cdot v_{j}^{I}\left(t\right)-f_{j}^{I}\left(t\right)\cdot v_{i}^{E}\left(t\right)-0.01\right).$$

As negative weights do not make physical sense in our implementation of the spiking neural network, at each time step we enforced the condition (W<0)=0 for all weights. Similarly, we enforced that $\left(W>W_{max}\right)=W_{max}$.

#### Single track experiment metrics

To evaluate the ability of a network with a given set of plasticity conditions, we ran 10 networks, each with a different set of random initial weights and different tuning centers for the input neurons, for 100 laps. To get a qualitative understanding, we split the track into 10 discrete locations and computed the spatial activity rate maps from the experiments using the last 25 laps (examples shown in Figure 1C top). To make the observations we derived from the rate maps quantitative, we also computed two different similarity metrics for each excitatory neuron every 10 laps (using *only* the activity from those 10 laps). Both have the form

$$S_{i}=1-\frac{{\bar{a}}_{i}^{max}-{\bar{a}}_{i}^{control}}{{\bar{a}}_{i}^{max}+{\bar{a}}_{i}^{control}}$$

where ${\bar{a}}_{i}^{max}$ is the average rate at the location with maximal activity and ${\bar{a}}_{i}^{control}$ is the average rate for a control location. For nearest neighbor similarity, the control locations are the positions on either side of the maximal location (circularly for boundary locations). For halfway similarity, we take the position on the track that is (circularly) the farthest from the location of maximal activity. The mean and standard error of the mean (SEM) for each of these metrics are plotted in Figure 1C bottom.

#### Two track experiments

To investigate how the spiking neural network was affected by exposure to a novel environment, and to see what role plasticity played in the encoding of the novel environment, we initialized and ran 10 networks under different plasticity conditions. For each experiment, we created two tracks, track A and track B, defined by their random input neuron tunings (i.e., T_i_), with the weights that connected the input to excitatory neurons (i.e., ${\text{W}}^{\text{InputE}}$) kept constant for both tracks. We trained a naive network on track A for 30 laps (the number of laps needed for the similarity metrics to reach a plateau), then transferred that network (that is, used the “learned” ${\text{W}}^{\text{EE}}$, ${\text{W}}^{\text{EI}}$, and ${\text{W}}^{\text{IE}}$) to either track A again, called A’, or to track B to compare the activity obtained from the re-exposure to the “same” environment, track A, to the exposure to a “different” environment, track B. This investigated how much of the difference between the activity on track A and track B was due to noise and the stochastic nature of the spiking network.

To examine the effects of plasticity and novelty quantitatively, we computed two metrics. The first was the Pearson correlation of the rate maps computed on the last 50% (15 laps) of the activity (Figure 1A left). We call this place field similarity, and the mean and SEM of the 10 simulations are plotted in Figure 1D, top, for each plasticity condition. The second metric is the Pearson correlation of the Kendall correlations computed on each excitatory neuron cell pairs, again computed on the last 50% of the activity (15 laps) (Figure 1A right). We call this cofiring similarity, and the mean and SEM of the 10 simulations are plotted in Figure 1D, bottom, for each plasticity condition. To compute Kendall correlations, we first split the activity into bins of length $\Delta t_{Kendall}=0.25$ and then averaged within each bin.

#### Parameters

The parameters used for the simulations are listed below.

**Table:** 

Parameter	Value	Description
n_input_	1000	# input neurons
n_excit_	500	# excitatory neurons
n_inhib_	50	# inhibitory neurons
p_input_	0.20	parameter of binomial distribution for number of tuning centers of input neuron
p_inputE_	1.0	parameter of binomial distribution for number of excitatory neurons each input neuron is connected to
p_EE_	0.25	parameter of binomial distribution for number of excitatory neurons each excitatory neuron is connected to
p_EI_	0.175	parameter of binomial distribution for number of inhibitory neurons each excitatory neuron is connected to
p_IE_	0.30	parameter of binomial distribution for number of excitatory neurons each inhibitory neuron is connected to
η	0.0005	synaptic learning rate
α	0.05	strength of pink noise
Δt	0.007	timescale of simulation
*r_PF_*	8	firing rate at tuning center
$\sigma^{2}$	0.1	width of tuning center
$\tau_{IF}$	0.02	absolute refractory period of excitatory and inhibitory neurons
$T_{IF}$	1	spiking voltage threshold
$\Delta t_{Kendall}$	0.25	size of bin used to average the activity before Kendall correlation
$W_{max}^{InputE}$	0.05	maximum initial weight between input neurons and excitatory neurons
*W_max_*	0.50	maximum value any weight can take

#### Histology

Mice were deeply anesthetized with Nembutal (100 mg/kg, i.p.) and perfused through the heart with cold saline followed by cold 4% paraformaldehyde (PFA) in PBS. The brains were removed, postfixed overnight in 4% PFA, and then cryoprotected in 30% sucrose for a minimum of 3 days. The brains were sectioned at 30–50μm, stained with DAPI, and examined with a fluorescence microscope.

## Supplementary Material

1

2

3

4

## Figures and Tables

**Table:** KEY RESOURCES TABLE

REAGENT or RESOURCE	SOURCE	IDENTIFIER
Bacterial and virus strains		
AAV1.Syn.GCaMP6f.WPRE.SV40	Addgene (Penn Vector Core)	https://www.addgene.org/100837/
Deposited data		
Calcium imaging and position data	This paper	https://doi.org/10.7910/DVN/L0Z9AK
Experimental models: Organisms/strains		
Mice with a mixed C57BL/6J background	Jackson Laboratory	https://www.jax.org/strain/000664
Software and algorithms		
MATLAB	MathWorks	http://www.mathworks.com/
JMP	JMP	https://www.jmp.com/
Prism	GraphPad	https://www.graphpad.com/features
Custom MATLAB analysis code	This paper	https://doi.org/10.7910/DVN/L0Z9AK
Custom Python analysis code	This paper	https://doi.org/10.7910/DVN/L0Z9AK

## Highlights

- Hippocampal CA1 place fields change randomly across environments, known as remapping
- Place field remapping only causes modest changes in short-timescale CA1 population cofiring
- Environment-specific cofiring patterns are smooth trajectories on the CA1 neural manifold
- ~20% of CA1 cells with the most anti-cofiring discharge relationships discriminate environments

## INCLUSION AND DIVERSITY

We worked to ensure sex balance in the selection of non-human subjects. One or more of the authors of this paper self-identifies as an underrepresented ethnic minority in their field of research or within their geographical location. One or more of the authors of this paper self-identifies as a gender minority in their field of research. One or more of the authors of this paper self-identifies as a member of the LGBTQIA+ community. While citing references scientifically relevant for this work, we also actively worked to promote gender balance in our reference list.
